# Development of a home-based training program for post-ward geriatric rehabilitation patients with cognitive impairment: study protocol of a randomized-controlled trail

**DOI:** 10.1186/s12877-017-0615-0

**Published:** 2017-09-12

**Authors:** Martin Bongartz, Rainer Kiss, Phoebe Ullrich, Tobias Eckert, Jürgen Bauer, Klaus Hauer

**Affiliations:** 1 AGAPLESION Bethanien Hospital Heidelberg, Geriatric Centre of the University of Heidelberg, Rohrbacher Str. 149, 69126 Heidelberg, Germany; 2 Department of Geriatrics, University of Heidelberg, 69117 Heidelberg, Germany

**Keywords:** Home-based training, Post-ward (patients) geriatric rehabilitation, Cognitive impairment, Physical activity promotion, Motor performance

## Abstract

**Background:**

Geriatric patients with cognitive impairment (CI) show an increased risk for a negative rehabilitation outcome and reduced functional recovery following inpatient rehabilitation. Despite this obvious demand, evidence-based training programs at the transition from rehabilitation to the home environments are lacking. The aim of this study is to evaluate the efficacy of a feasible and cost-effective home-based training program to improve motor performance and to promote physical activity, specifically-tailored for post-ward geriatric patients with CI.

**Methods:**

A sample of 101 geriatric patients with mild to moderate stage CI following ward-based rehabilitation will be recruited for a blinded, randomized controlled trial with two arms. The intervention group will conduct a 12 week home-based training, consisting of (1) Exercises to improve strength/power, and postural control; (2) Individual walking trails to enhance physical activity; (3) Implementation of patient-specific motivational strategies to promote behavioral changes. The control group will conduct 12 weeks of unspecific flexibility exercise. Both groups will complete a baseline measurement before starting the program, at the end of the intervention, and after 24 weeks for follow-up. Sensor-based as well as questionnaire-based measures will be applied to comprehensively assess intervention effects. Primary outcomes document motor performance, assessed by the Short Physical Performance Battery, and level of physical activity (PA), as assessed by duration of active episodes (i.e., sum of standing and walking). Secondary outcomes include various medical, psycho-social, various PA and motor outcomes, including sensor-based assessment as well as cost effectiveness.

**Discussion:**

Our study is among the first to provide home-based training in geriatric patients with CI at the transition from a rehabilitation unit to the home environment. The program offers several unique approaches, e.g., a comprehensive and innovative assessment strategy and the integration of individually-tailored motivational strategies. We expect the program to be safe and feasible in geriatric patients with CI with the potential to enhance the sustainability of geriatric rehabilitation programs in patients with CI.

**Trial registration:**

International Standard Randomized Controlled Trial (#ISRCTN82378327). Registered: August 10, 2015.

## Background

The prevalence of cognitive impairment (CI) in patients admitted to geriatric rehabilitation units ranges from 30% to 80%, depending on sample characteristics and cut-off criteria [1, 2]. CI is accompanied with high demands for the health care systems, as decreased cognitive functioning is associated with increased care costs in this vulnerable population [3]. Compared to cognitively-intact patients, patient with CI show an increased number of risk factors affecting their health status, i.e., multi-morbidity [4], lower functional status [5], an increased risk of falling [6], and higher institutionalization and mortality rates [2]. Particularly, CI is often associated with specific motor-related symptoms, such as impaired functioning (i.e., deficits in balance and gait performance) [7–9], reduced participation in activities of daily living (ADL; i.e., shopping, dressing, or eating) [9] and reduced outdoor activities [10]. Further, a higher probability of neuropsychiatric symptoms is typical in patients with CI (i.e., apathy, anxiety, depression, irritability and agitation) [11–13], resulting in a loss of motivation to become physically active [14]. Patients with CI show an increased risk for negative rehabilitation outcome, leading to limited functional recovery during inpatient rehabilitation [15] and a lower functional status at hospital discharge [5]. Also, their access to medical services is limited, including post-ward rehabilitation (e.g., traveling too far to participate in rehabilitation programs is considered a typical barrier) [16]. These findings indicate the need for appropriate rehabilitation concepts for geriatric patients with CI at the transition from inpatient rehabilitation to their home environments and innovative, individually-tailored training concepts with low entry barriers are required. Effective post-ward rehabilitation programs may increase the ability to perform ADL, representing one of the most important predictor of societal costs of care of community-dwelling patients and improve functional performances and mobility (i.e., allowing proper preservation of autonomy).

Home-based training programs represent suitable exercise regimens that have been shown feasible and save in community-dwelling older adults with CI [17–21]. However, results of these home-based training programs are inconsistent. Some studies indicated improvements in motor performance [17, 18], while others only showed decelerated deterioration of physical function and mobility [22] or did not find any effects on performance [20, 21]. Further, studies showed methodological inconsistencies, e.g., not providing sufficient information on registered motor performance measures [20], not measuring key motor features (i.e., gait, balance, strength) as primary endpoints [19, 20], or using rather small sample sizes [18, 21].

Publications on home-based training programs in the vulnerable group of post-ward patients with CI are still scarce or inconclusive, despite overwhelming evidence based on supervised ward-based and post-ward programs are effective in improving motor function and physical activity (PA) in these specific population [23, 24]. Interventions at the transition from geriatric inpatient rehabilitation to the home environments have only been evaluated in heterogeneous patient populations [25–29]. Most programs included cognitively intact patients [25, 26] and only few studies integrated sub-groups of cognitively-impaired patients [27–29]. Some studies reported beneficial effects of home-based training on motor performance and mobility [25, 29], while others did not find training-related performance improvements [26, 27]. Furthermore, Moseley and colleagues indicated that patients with CI showed worse adherence rates compared to cognitive-intact persons during a home-based training program [27]. Hence, motivational strategies should be integrated into physical exercise programs to promote adherence and increase participation. The importance of motivational strategies is further emphasized by Heyn and colleagues [30], who explicitly state that previous interventions did not integrate appropriate motivational concepts to foster participation and enhance motivation in older adults with CI.

Surprisingly, behavioral aspects, such as PA have hardly been investigated in home-based training programs, although such behavioral changes are crucial for the sustainability of rehabilitation programs. Primary aim of most programs was to improve motor performance and only a few studies documented PA using questionnaires [17, 18, 21]. None found training-related improvements and none objectively registered PA using sensor-based assessment strategies. The latter is astonishing since, due to recall and response bias, questionnaire-based assessments are less valid and reliable in cognitively-impaired older adults [31]. Thus, assessment strategies including objective sensor-based as well as subjective questionnaire-based measurements are advised to comprehensively register changes in activity behavior following training interventions.

The current study protocol addresses the situation of multi-morbid and frail geriatric patients with mild to moderate CI following inpatient rehabilitation. Primary aim of the study is the development and evaluation of a safe and feasible home-based exercise program to improve motor performance and to promote PA (i.e., initiate sustained behavioral changes) in the vulnerable group of multi-morbid, geriatric patients with CI, suitable to be implemented in existing health care plans.

## Methods/Design

### Study design

**Fig. 1:**
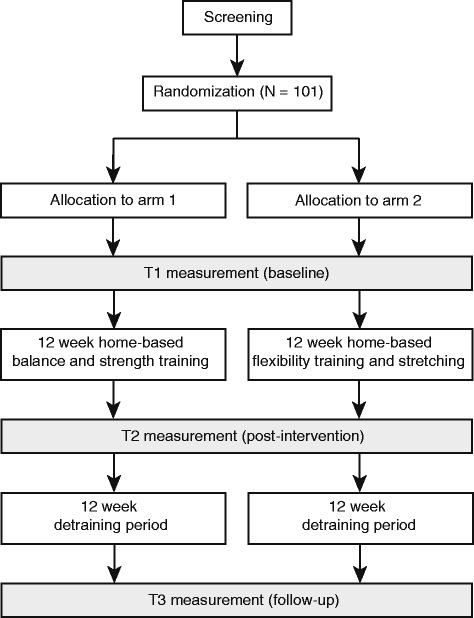
Schematic illustration of the study design

The presented study is a blinded randomized-controlled trial (RCT) with two arms (i.e., an intervention group [INTV] and a control group [CTRL]; Fig. 1) and will be implemented according to CONSORT guidelines [32]. Ethical approval according to the ethical standards of the Helsinki declaration was obtained from the internal review board at the Medical Faculty of the University of Heidelberg, Germany (reference#: S-252/2015). The study protocol has been registered with the “International Standard Randomized Controlled Trial Number” (ISRCTN) trial register (http://www.isrctn.com/ISRCTN82378327).

### Sample characteristics

Inclusion criteria are (1) patients admitted to ward-based rehabilitation in a geriatric hospital in Heidelberg, Germany; (2) age ≥ 65 years; (3) mild to moderate CI (Mini-Mental-State-Examination [MMSE]: 17–26); (4) ability to walk 4 m without support; (5) residency within 30 km of the study center; (6) discharge from rehabilitation unit to home environment; (7) no terminal disease/no delirium; (8) German-speaking; and (9) written informed consent from participant or care giver. All patients admitted to the geriatric inpatient rehabilitation at the AGAPLESION Bethanien Hospital are consecutively screened for inclusion.

### Measures

After inclusion of the participants, primary and secondary outcome parameters are registered at baseline (T1), following the training intervention (T2), and 12 weeks after the training is completed (T3). At baseline, additional descriptive parameters (i.e., age, gender, height, weight, Barthel Index) are registered. Blinded assessors will conduct the measurements at the patients’ home.

### Motor performance

Key motor performances will be assessed using the Short-Physical-Performance-Battery (SPPB) including sub-tests of static balance, walking performance and sit-to-stand performance [33]. Outcome parameters are the total SPPB score (i.e. 0–12 scores) calculated as the summation of each sub-test score and the scores in each of the sub-tests (i.e. 0–4 scores) [33]. The “Timed Up and Go-test” (TuG) is performed to assess basic functional mobility [34]. Outcome parameter is the time in seconds needed to perform the task.

Additionally, objective performance measures are registered for SPPB using advanced body-fixed motion sensors (DynaPort MT, McRoberts B.V., The Hague, Netherlands). The sensor is attached to subject’s lower back at the height of the second lumbar vertebra. The measurement system (106.6 × 58 × 11.5 mm, 55 g) contains three pre-calibrated seismic accelerometers (sensor range: ± 2 g, resolution: 1 mg) and three gyroscopes (range: ± 100 deg./s, resolution: 0.0069 deg./s). Data is recorded at a sampling rate of 100 Hz. Outcome parameters are defined as body sway in terms of Center of Pressure (CoP) displacements in mm and CoP velocity in mm/s to evaluate static balance performance. During the 5-times-chair-rise test, sit-to-stand and stand-to-sit durations in seconds and maximum angular velocities in deg./s are calculated.

### Physical activity

PA will be registered using a small-scaled (51 × 30 × 16 mm, 24 g) activity monitor (PAMSys, BioSensics, Cambridge, MA, USA), attached on subjects’ chest using adhesive bands. The activity monitor includes a tri-axial accelerometer sensor registering accelerations in three perpendicular directions (accelerations in the frontal, vertical, and lateral directions) at a sampling frequency of 40 Hz. Additionally, a second activity monitor (uSense) will be used for validation purpose. The uSense sensor is a non-commercial prototype, developed by the EU-funded “FARSEEING” project allowing detailed quantitative as well as qualitative data analysis. The sensor (42 × 10 × 68 mm, 36 g) includes a 9-axis inertial platform (accelerometer, gyroscope, magnetometer) registering acceleration and orientation in X, Y and Z direction at a sampling frequency of 100 Hz and is attached to subjects’ lower back using adhesive bands.

Raw data from both activity monitors is transferred to a stationary computer for offline analysis using established algorithms [35]. Outcome parameters are number and duration (registered in minutes) of postural episodes (lying, sitting, standing, and walking), cadence defined as steps per minute, number of steps (i.e., total number of steps, number of steps per gait episode) and sit-to-stand/stand-to-sit transitions (number of sit-to-stand/stand-to-sit transitions). The uSense sensor additionally registers qualitative gait parameters (e.g., mean step duration [s], step regularity [%], turning velocity [deg/s]) during each walking episode. Participants are asked to wear the monitor continuously for 48 h.

### Life-space

The University of Alabama at Birmingham Study of Aging Life-Space Assessment has been used to document the mobility of community-dwelling older people [36]. Life-space zones range from a person’s bedroom (level 0) to beyond the person’s home town (level 5). For each life-space zone, subjects report how often they travel to that area per week and whether they need assistance. Higher scores indicate high life-space mobility from 0 (“totally bed-bound”) to 120 points (“traveled out of town every day without assistance”) [36]. We developed a modified version of the University of Alabama at Birmingham Study of Aging Life-Space Assessment to assess participants’ life-space (LSA). The LSA is adjusted to specific limitations of patients with CI to eliminate recall bias and has been successfully validated in geriatric patients with CI (paper submitted).

Additionally, participants’ outdoor life-space will be objectively measured using a Global Position System (GPS) tracker. Participants are asked to wear the mobile GPS tracker (QStarz BT1000X, Qstarz International Co., Ltd., Taipei, Taiwan) for 48 consecutive hours. Using a sampling rate of 0.2 Hz, the GPS tracker records participants’ outdoor location (i.e., longitude and latitude coordinates) with an accuracy of ±5 m. Outcome measures for a patient’s life-space are total LSA score, the maximum distance in meters from the participant home and the mean distance of outdoor walking episodes in meters assessed by GPS using location data [37].

### Psycho-social parameters

#### Health-related quality of life – EuroQol - 5 Dimensions (EQ-5D):

The EQ-5D, including five health-related domains (mobility, self-care, pain/discomfort, usual activities and anxiety/depression) will be used to measure health-related quality of life [38]. Outcome parameters are the EQ-5D total score (5–15 points) and participants’ self-rated perceived health status (0–100 points).

#### Geriatric Depression Scale – Short Form (GDS-SF):

The 15-item GDS-SF will be used to register depressive symptoms [39] by assessing their presence/absence in frail older people (e.g. “Are you basically satisfied with your life?” or “Do you feel happy most of the time?”) [40]. A total score of 0–15 points can be achieved with higher scores representing a higher probability of depression (i.e., total score > 5 points indicates mild depressive symptoms, a total score ≥ 10 points indicates moderate to severe depression) [39].

#### Apathy Evaluation Scale-Clinical version (AES-C):

The 18 item AES-C will be used to evaluate participants’ for apathy [41]. The AES-C defines apathy as a psychological dimension based on deficits in behavioral, cognitive, and emotional circumstances of goal-directed behavior. Using the AES-C, the evaluation of apathy is based on clinical observations and subjects’ self-reports during a semi-structured interview. A total score of 0 to 54 points can be achieved with higher scores representing a higher probability of apathy.

### Fall-related parameters

#### Fall history:

Falls are defined as an unexpected event where a person comes to rest on the ground, floor or lower level [42]. We will assess the number of falls prospectively during the intervention period using diaries administered by the participants and weekly phone calls conducted by the trainer [43]. Fall history of patients is registered retrospectively at T1, T2 and T3 by asking the participants how often he/she fell during the preceding 4 weeks and 12 months. At T3, participants are additionally asked how often he/she fell during the preceding 12 weeks between T2 and T3.

#### Short - Falls Efficacy Scale – International (Short-FES-I):

Fall-related self-efficacy will be registered using the 7-item Short-FES-I [44], measuring the level of concern about falling during indoor/outdoor social and physical activities. A total score of 7 (no concern about falling) to 28 (severe concern about falling) can be achieved.

#### Fear of Falling Avoidance Behavior Questionnaire (FFABQ):

The FFABQ is used to assess activity avoidance behavior due to fear of falling [45]. The FFABQ total score ranges from 0 to 56 points. Higher score indicate greater activity limitations and participation restrictions as a consequence of fear of the falling.

#### Cognitive Performance

The Digit-Span sub-test of the Wechsler Adult Intelligence Scale (WAIS) [46] is registered to assess participants’ short-term working memory. The number of correct repetitions is used for further analyses.

### Cost effectiveness

To evaluate cost effectiveness, incremental cost-effectiveness ratios (ICERs) will be calculated [47]. ICERs are calculated by dividing the difference in costs (between the INTV and the CTRL) by the group difference in the outcomes and can be interpreted as the cost to obtain an extra unit of effectiveness, quantifying the trade-offs between patient outcomes gained and resources spend [47]. In our analysis, ICERs will be calculated for primary outcomes (e.g., *motor performance; SPPB total score*) and secondary outcomes (e.g., *health-related quality of life; EQ-5D questionnaire*). Therefore, average costs of the exercise interventions (e.g., training material, visits by trainer, phone calls) as well as overall health care costs (e.g., general practitioner consultations, inpatient and outpatient care, days in hospital, hours of nursing care, hours of professional domestic help or help by family members/friends) will be collected for INTV and CTRL [48]. Overall health care costs will be assessed retrospectively, covering a period of 12 weeks before T1, T2 and T3, using an established questionnaire (FIMA) [49].

### Primary outcomes

- *motor performance:* SPPB total score (0–12 points)
- *physical activity:* duration of active episodes (i.e., sum of standing and walking)

### Secondary outcomes

- *motor performance:* SPPB sub-test scores (0–4 points each), TuG total time, sensor-based parameters during SPPB sub-tests (e.g. CoP displacements, CoP velocity, sit-to-stand and stand-to-sit duration, maximum angular velocities), and qualitative gait parameters (e.g., mean step duration, step regularity, turning velocity)
- *physical inactivity:* duration of inactive episodes (i.e., sum of lying and sitting), quantitative gait parameters (i.e., cadence as steps per minute, total number of steps, and number of steps per gait episode)
- *life-space:* LSA total score (0–90 points), maximum distance from home and mean walking distances (GPS-based measurements)
- *quality of life:* EQ-5D total score (5–15 points), VAS score (0–100 points)
- *psychosocial status:* GDS-SF total score (0–15 points), AES-C total score (0–54 points), FES-I total score (7–28 points), FFABQ score (0–56 points)
- *falls*: fall history, number of falls during the intervention period
- *short-term working memory:* WAIS-IV test performance (0–9 points)
- *cost evaluation:* ICER for operational as well as health care costs

### Intervention

**Fig. 2:**
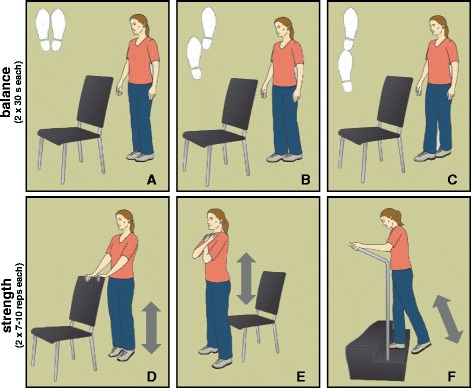
Illustration of the exercise provided on a poster and used in the intervention. The upper row displays balance exercise (**a**-**c**), the lower row represents strength exercises (**d**-**f**)

Participants in INTV take part in a standardized 12-week home-based training program. The program is specifically-tailored for geriatric patients with CI after post-ward rehabilitation to improve motor performance and enhance PA. The training intervention was derived from an exercise program developed by our research group, which was feasible and effective to increase strength and functional performances in patients with CI [24]. Based on results of a successfully conducted home-based exercise pilot RCT, our program includes six exercises to improve static and dynamic postural control (i.e., standing and walking) and strength (i.e., tiptoe stance, stair climbing, sit-to-stand transfers). Exercises are explained and regularly reviewed by a professional trainer and illustrated at the patient’s home using a large poster (84.1 × 59.4 cm; Fig. 2). A brief description of key elements for each exercise is provided using a printed manual. Training progress and adherence is supervised by weekly phone calls and regular home visits of the trainer (5 visits in 12 weeks).

One major aim of the intervention is to motivate participants to induce behavioral changes in terms of enhanced PA by incorporating walking trails into their everyday life. Therefore, an individual outdoor walking course is defined next to the patients’ homes (i.e., in the respective neighborhoods). Motivational strategies will be used to encourage behavioral changes. Participants are asked to regularly set individual goals, e.g., to extend the individually-defined walking distance. Barriers hampering regular walking sessions and solutions to overcome these barriers are identified in cooperation with the trainer and benefits of regular training/walking sessions are discussed. Variations of the walking trails and physical exercises program are developed to adjust the training protocol to meet the participants’ needs (e.g., number of repetitions, handrail support, eyes open/closed, etc.). During the home visits and weekly phone calls, the trainer encourages the participant to conduct the training exercises and the walking trails independently. Furthermore, training descriptions (i.e., training manual; poster), training logs and pedometers are provided to foster participation, to enable self-monitoring of performance, and to define individual goals (e.g., increase number of daily steps). Participation in a local sports club is offered to each participant as an incentive for social participation.

The training program will include a motivational concept that is based on the Theory of Planned Behavior (TPB [50]), Social-Cognitive Theory (SCogT [51]), and Control Theory (CT [52]). The concept will use behavioral change techniques introduced by Abraham & Michie (cf. [53] for a detailed description of the theoretical framework) and integrated into our intervention as followed:

1. Information about benefits/consequences of regular training and walking sessions are provided (based on TPB);
2. Encouragement to change daily routines (i.e., integrate training, walk regularly, join a local sport club) (based on TPB);
3. Barrier identification that hamper training and development of solutions to overcome barriers (based on SCogT);
4. Set graded tasks (i.e., variations of exercises and walking trails are developed) (based on SCogT);
5. Provide specific instructions (during home visits using poster and manuals) (based on SCogT);
6. Definition of training goals (i.e., frequency, intensity and duration of exercises and walking trails) (based on CT);
7. Review of behavioral goals (home visits, regular phone calls, pedometer) (based on CT);
8. Self-monitoring of behavior (training logs, pedometer) (based on CT);
9. Provide feedback to reinforce behavioral change (home visits, phone calls, pedometer) (based on CT/positive).

These behavioral change techniques are used to encourage patients to translate their intentions into behavioral change. Participants are expected to develop intrinsic motivation and volition to adapt their behavior.

Participants in CTRL receive newsletter-based information about unspecific flexibility and strength training, nutrition and relaxation over a period of 12 weeks. Similar to INTV, participants are called weekly and receive the same amount of home visits as the INTV group to exclude bias effects based on social support. After finishing the project, participants in CTRL will have the opportunity to perform the same exercise program than INTV and also join the local sports club.

### Statistical analysis and sample size

An intention to treat analysis will be conducted. We performed an a priori power analysis to determine the sample size necessary to obtain significant effects in PA. Power analysis for PA is difficult to calculate since studies using identical sensor-based physical activity assessments in cognitively-impaired geriatric patients have not been available. Thus, the effect size is based on a study conducting a functional strength training program in 137 institutionalized patients [54]. PA, as assessed by the MTI Actigraph, showed an average increase of PA by 43% in the strength training group compared to a control group (effect size: eta^2^ = 0.18). A priori power analysis was performed using GPower 3.1 software [55]. A repeated measure analysis of co-variance (ANCOVA) design including two groups and two repeated measurements yielded a total sample size of *N* = 101 (α = 0.05; critical F = 2.69). Estimating a drop out of 15%, we will recruit a total of 116 participants. For motor performance, as assessed by the SPPB total score, a pilot study conducted by our research group showed that performance improvements can be achieved using relatively small sample sizes (*N* = 34; large effect size: eta^2^ = 0.22 [56]). Repeated measures ANCOVA will be used to determine effects of the home-based training program on primary and secondary outcomes for effects of intervention (T1-T2) and sustainability of effects (T1-T3). Effect sizes are determined by calculating eta^2^ [57]. All data will be analyzed using SPSS 23.0 (SPSS Inc., Chicago, Ill., USA).

## Discussion

Our study is among the first RCTs to investigate a specifically-tailored home-based training program with low entry barriers in geriatric patients with CI at the transition from a rehabilitation unit to their home environment. The proposed training program may represent a feasible and effective tool to improve motor performance and increase PA in patients with CI, thus fostering a sustained change in patients’ activity behavior. The program requires relatively low supervision and material costs and has the potential to be implemented into existing health care plans. If the program will prove to be effective, it may lower the barrier for post-ward geriatric patients to take up training and exercising. The program may enhance the sustainability of rehabilitation programs by improving medical care of multi-morbid geriatric patients with CI using an innovative therapeutic concept at this vulnerable stage of health care.

Previous home-based training studies showed methodological limitations, e.g., only included sub-groups of patients with CI [27–29] or did not measure key-motor performances as primary endpoints [25, 28] and behavioral aspects in terms of PA have not been investigated in geriatric patients with CI. Only a few studies investigated PA in community-dwelling older adults with CI using questionnaires [17, 18, 21]. Hence, there is a lack of studies in post-ward geriatric patients comprehensively evaluating motor performance and PA. The investigation of training-related effects on motor performance and behavioral changes in terms of enhanced PA requires innovative assessment strategies, including sensor-based as well as questionnaire-based measures. In the presented study we will assess primary outcome measures using well-established, validated tests and complement this approach by additionally registering objective and detailed, sensor-based motor data, a range of psycho-social parameters, such as fear of falling, depression and apathy as well as participants mobility status (life-space), quality of life and cognitive functioning. Applying this comprehensive assessment strategy, we will be able to identify training-related modifications of PA and motor performance based on state-of-the-art sensors and examine their relationship with potentially influencing factors, among others, cognitive status or fear of falling. We regard our assessment as a unique and comprehensive combination of quantitative, qualitative, questionnaire and sensor-based data that - to our knowledge - is not available so far.

One aim of our study is to modify behavior in terms of enhanced PA. Participants need to develop intrinsic motivation and volition to adapt their behavior and to become physically active. So far, only one home-based training study for older adults with CI implemented motivational strategies to increase adherence to physical training, by integrating a goal-oriented and individually-tailored training according to the individual’s needs [22]. However, authors did not describe the specific motivational strategy used and did not show data on the feasibility of the motivational support. Our study is going to address this limitation by integrating a specific motivational strategy for patients with CI. Based on various theoretical frameworks (i.e., TPB, SCogT, and CT) [50–52], we will integrate several behavioral change techniques (i.e., information on consequences, barrier identification, proper instructions, goal setting, review of behavioral goals, self-monitoring, and performance-based feedback [53]) into our intervention to ramp up adherence and to increase PA. To the authors knowledge, such a comprehensive interventional approach aiming at behavioral modifications by using individually-tailored motivational instruments not been implemented and evaluated in a home-based training program for patients with CI at the transition from rehabilitation to their home environment before.

In addition, CI represents a high financial burden for the health care systems in western societies [3]. Limited resources in health care require effective interventions that provide high benefits relative to the costs [58]. The presented home-based training program aims to counteract typical health-related limitations in patients with CI and represents a low budget approach. In line with this, our program may encourage the participants to conduct the training sessions independently at their home environments (i.e., no professional monitoring) to minimize costs for supervision by professional trainers. To our knowledge, our study will be the first to also assess economical aspects of a home-based training program for geriatric patients with CI using established instruments (i.e., FIMA [49]) and analyses (i.e. ICER [47, 48]) to objectively yield potential cost effectiveness of the intervention.

In summary, this trial will provide insight into the effect of a specifically-tailored home-based exercise program in patients with CI on motor performance and behavioral change. The program offers several approaches, e.g., a comprehensive and innovative assessment strategy and the integration of individually-tailored motivational strategies to stimulate behavioral change and increase motor performance, may have the potential to enhance the sustainability of geriatric rehabilitation programs in patients with CI.

## Abbreviations

ADL: Activities of daily living
AES-C: Apathy evaluation scale-clinical version
CI: Cognitive impairment
CoP: Center of pressure
CT: Control theory
CTRL: Control group
deg.: Degrees
EQ-5D: EuroQol - 5 Dimensions
FFABQ: Fear of falling avoidance behavior questionnaire
GDS-SF: Geriatric Depression Scale – Short Form
GPS: Global position system
ICERs: Incremental cost effectiveness ratios
INTV: Intervention group
LSA: Life-space assessment
MMSE: Mini-Mental-State-Examination
PA: Physical activity
RCT: Randomized-controlled trial
SCogT: Social-cognitive theory
Short-FES-I: Short - Falls Efficacy Scale – International
SPPB: Short-Physical-Performance-Battery
TPB: Theory of planned behavior
TuG: Timed up and go
WAIS: Wechsler adult intelligence scale
